# Peripheral β-Endorphin in Borderline Personality Disorder: Investigating Clinical Correlates and Longitudinal Changes During Psychotherapy

**DOI:** 10.3390/biology15211912

**Published:** 2026-10-22

**Authors:** Elisabetta Maffioletti, Chiara Galbiati, Elena Milanesi, Andrea Geviti, Daniela Tardito, Mariangela Lanfredi, Laura Pedrini, Serena Meloni, Antonino Carcione, Giuseppe Nicolò, Antonio Semerari, The CLIMAMITHE Study Group, Roberta Rossi, Luisella Bocchio-Chiavetto

**Affiliations:** 1 Department of Theoretical and Applied Sciences (DiSTA), eCampus University, 22060 Novedrate, Como, Italy; elisabetta.maffioletti@uniecampus.it (E.M.); daniela.tardito@uniecampus.it (D.T.); 2 Psychiatry Unit, IRCCS Istituto Centro San Giovanni di Dio Fatebenefratelli, 25125 Brescia, Italy; mlanfredi@fatebenefratelli.eu (M.L.); lpedrini@fatebenefratelli.eu (L.P.); smeloni@fatebenefratelli.eu (S.M.); rrossi@fatebenefratelli.eu (R.R.); 3 Biological Psychiatry Unit, IRCCS Istituto Centro San Giovanni di Dio Fatebenefratelli, 25125 Brescia, Italy; c.galbiati@fatebenefratelli.eu; 4 Victor Babes National Institute of Pathology, 050096 Bucharest, Romania; elena.k.milanesi@gmail.com; 5 Faculty of Medicine, Carol Davila University of Medicine and Pharmacy, 050474 Bucharest, Romania; 6 Service of Statistics, IRCCS Istituto Centro San Giovanni di Dio Fatebenefratelli, 25125 Brescia, Italy; ageviti@fatebenefratelli.eu; 7 Third Centre of Cognitive Psychotherapy, 00161 Rome, Italy; nino.carcione@gmail.com (A.C.); giuseppe.nicolo@aslroma5.it (G.N.); semerari@terzocentro.it (A.S.); 8 Department of Human Sciences, Guglielmo Marconi University, 00193 Rome, Italy

**Keywords:** borderline personality disorder, β-endorphin, biomarker, non-suicidal self-injury, psychotherapy, treatment response, metacognitive interpersonal therapy, structured clinical management

## Abstract

Borderline personality disorder (BPD) is characterized by emotion dysregulation, interpersonal dysfunction, and frequent non-suicidal self-injury (NSSI). Altered pain sensitivity in BPD has raised interest in the possible role of the endogenous opioid system, but only a few studies have explored β-endorphin, one of the major endogenous opioid peptides, in this disorder. Here we investigated serum β-endorphin levels in patients with BPD, their association with clinical features, and their longitudinal changes during psychotherapy. Forty-nine patients with BPD enrolled in the CLIMAMITHE randomized controlled trial and 25 age- and sex-matched unaffected controls were included. Serum β-endorphin concentrations were measured by ELISA at baseline in all participants and also after 6 and 12 months of treatment in patients receiving metacognitive interpersonal therapy or structured clinical management. Baseline β-endorphin levels did not differ between patients and controls and were not associated with symptom severity, interpersonal functioning, attachment style, psychotropic treatment, or NSSI. No overall longitudinal changes were observed during psychotherapy. However, responders showed a significant increase in β-endorphin levels from T0 to T12 (*p* = 0.016), whereas non-responders remained unchanged. Moreover, responders exhibited significantly lower baseline β-endorphin concentrations than non-responders (*p* = 0.001), and baseline levels showed potential discriminative value for treatment response (AUC = 0.806, *p* = 0.002) and correlated with changes in emotion dysregulation during treatment, measured through the DERS (r = 0.344, *p* = 0.040). In conclusion, these findings suggest that peripheral β-endorphin is unlikely to represent a trait biomarker of BPD but may be associated with response to psychotherapy, although its potential clinical relevance requires validation in larger independent cohorts.

## Simple Summary

Borderline personality disorder is characterized by difficulties in regulating emotions and managing relationships and is frequently associated with self-injury. Research has suggested that endogenous opioids could play a role in this last aspect, but so far little is known. This study examined the blood levels of β-endorphin, the main endogenous opioid, in 49 patients with borderline personality disorder and 25 healthy individuals. It also explored whether these levels were related to clinical characteristics and changed during one year of psychotherapy. Before the therapy, β-endorphin levels were similar in patients and unaffected people and were not linked to self-injury or other clinical features. Levels of β-endorphin also did not change overall during the psychotherapy. However, patients who improved had lower β-endorphin levels at the beginning and showed an increase during the treatment, unlike those who did not improve. If confirmed, these findings suggest that β-endorphin levels may help identify patients who are more likely to benefit from psychotherapy. Further research involving larger groups of participants is needed to confirm these results and determine whether measuring β-endorphin could eventually help to guide treatment and to improve care for borderline personality disorder.

## 1. Introduction

Borderline personality disorder (BPD) is a severe mental disorder characterized by emotion dysregulation, unstable identity, and disturbed interpersonal function.

The symptoms of BPD include acute dysfunctional behaviours, such as repetitive self-harm, chronic suicidal thoughts or attempts, aggressive outbursts and high sensitivity to rejection. Clinical manifestations of BPD can be extremely heterogeneous: patients may exhibit very different presentations, some predominantly showing heightened impulsivity, emotion dysregulation and affective instability, which attract more professional attention and urgency to intervene, whereas others present a clinical profile characterized by hyperarousal, emotion overmodulation and dissociative features. BPD symptoms first appear in early adolescence, peak in late adolescence and early adulthood and decline thereafter, and they can markedly vary both qualitatively and quantitatively over the life span. BPD is associated with a range of poor outcomes, including low occupational and educational achievement, lack of long-term close relationships and increased conflicts with partners, sexual risk-taking, and low levels of social support, leading to poor life satisfaction [1].

Nonetheless, BPD is not as untreatable as previously thought. Psychotherapy represents the first-line treatment, and pharmacotherapy can be used as adjunctive treatment in specific situations. Different psychotherapeutic approaches have demonstrated efficacy in treating BPD patients [2]. Among these, metacognitive interpersonal therapy (MIT) and structured clinical management (SCM) have been tested as treatments for BPD in a randomized controlled clinical trial conducted by our research group (CLIMAMITHE project) [3,4]; the results showed that both therapies produced significant improvements in BPD symptomatology [4].

From an etiopathogenetic perspective, the mechanisms underlying BPD are thought to result from a complex interplay of genetic, neurobiological, and environmental factors [5]. Among the biological processes implicated in the disorder, the most extensively investigated involve dysregulation of the hypothalamic–pituitary–adrenal (HPA) axis [6] and alterations in the oxytocin system [7,8,9]. These systems play a central role in stress response, emotion regulation and interpersonal functioning, all of which represent core domains of impairment in BPD.

Another biological mechanism that is attracting increasing attention is related to the endogenous opioid system. Interest in this system stems from evidence indicating altered pain sensitivity and processing in patients with BPD [10]. These alterations have been particularly investigated in relation to non-suicidal self-injury (NSSI), a core behavioural feature of BPD. NSSI consists of the deliberate and direct destruction of body tissue without suicidal intent and is thought to represent a maladaptive strategy for regulating intense negative emotional states and reducing aversive internal tension [11,12,13,14]. Most BPD patients report little or no pain during episodes of NSSI, and several studies have demonstrated reduced pain sensitivity in BPD patients who engage in NSSI compared with unaffected controls [14]. Specifically, BPD patients report less pain when exposed to thermal [15,16,17], electrical [18], mechanical and chemical stimuli [19]. Notably, lower pain sensitivity has been associated with higher levels of aversive internal tension and dissociative symptoms, suggesting a close relationship between altered pain processing and core symptoms of BPD [18].

The mechanism proposed to underlie the reduced pain sensitivity observed in patients with BPD may involve a dysregulation of the endogenous opioid system, which may result from histories of trauma and chronic stress and/or from a genetic predisposition. Within this framework, NSSI may represent a maladaptive coping strategy aimed at stimulating endogenous opioid release [20,21]. Moreover, neuroimaging studies have demonstrated altered baseline μ-opioid receptor availability and abnormal endogenous opioid system activation during negative emotional states in individuals with BPD [22].

β-endorphin is one of the major endogenous opioids and mainly acts as an agonist at μ-opioid receptors. It plays a key role in stress-induced analgesia and in the modulation of thermal pain perception. Interestingly, β-endorphin and adrenocorticotropic hormone (ACTH), the pituitary hormone involved in the stress response, share a common precursor, pro-opiomelanocortin (POMC) [23]. A limited number of studies have reported reduced basal β-endorphin levels in individuals exhibiting NSSI. A study conducted in adults with cluster B personality disorders, most of whom had BPD, described lower central β-endorphin levels, measured in cerebrospinal fluid (CSF), in patients with NSSI compared with those without [21]. In contrast, other studies performed in adolescents have examined peripheral β-endorphin levels, reporting lower plasma concentrations in individuals with NSSI compared with a control group [24] and reduced plasma β-endorphin in female adolescents with NSSI compared with controls [25]. However, a more recent study, also involving adolescent participants, found no association between peripheral (plasma) β-endorphin levels and either NSSI or BPD symptoms [26]. Overall, knowledge in this field remains limited.

Based on these findings and the current gaps in the literature, the present study aimed to further elucidate the role of β-endorphin in BPD by investigating its peripheral serum levels in affected individuals and their possible associations with clinically relevant features, including NSSI and emotion dysregulation. We also aimed to explore whether psychotherapy, consisting of MIT and SCM, is associated with changes in β-endorphin concentrations and whether these changes are related to treatment outcomes. As an exploratory objective, we also examined whether baseline β-endorphin levels are associated with subsequent treatment outcomes, as a potential predictor of psychotherapy response.

## 2. Materials and Methods

### 2.1. Study Participants

BPD patients and unaffected volunteers (controls, CTRL) were enrolled in the framework of the CLIMAMITHE project (ClinicalTrials.org: NCT02370316), a randomized controlled clinical trial evaluating the effects of MIT and SCM on clinical, neuroimaging and molecular outcomes. The trial was carried out at two centres: IRCCS Istituto Centro San Giovanni di Dio, Brescia, Italy (site 1; coordinating centre); Third Center of Cognitive Psychotherapy, Rome, Italy—Scuola Italiana di Cognitivismo Clinico (SICC), Rome, Italy (site 2). Unaffected controls were recruited separately and included as a reference group. The complete description of the clinical trial protocol and clinical results have been previously published [3,4]. The study protocol was approved by the Ethics Committee of the coordinating centre, IRCCS Istituto Centro S. Giovanni di Dio Fatebenefratelli, Brescia, Italy (Protocol No. 67/2014).

The inclusion criteria for patients were: (a) age between 18 and 45 years; (b) a diagnosis of BPD according to DSM-IV-TR; (c) signed informed consent. The exclusion criteria were: (a) a lifetime diagnosis of schizophrenia, schizoaffective disorder or bipolar disorder; (b) substance abuse or dependence in the 3 months before the enrolment; (c) organic mental syndromes, dementia or cognitive impairment; (d) relevant neurological signs; (e) in women, pregnancy or lactation; (f) concurrent psychotherapies. The inclusion criteria for CTRL were: (a) age between 18 and 45 years; (b) signed informed consent, whereas the exclusion criteria were any cognitive impairment or psychiatric/neurologic condition, including alcohol or substance abuse.

Within the overall CLIMAMITHE cohort, a subgroup of participants agreed to undergo blood sampling for molecular analyses; the present study included all BPD patients and CTRL with available serum samples, comprising 49 BPD patients and 25 age- and sex-matched CTRL. Blood samples from patients were collected at baseline (T0), after 6 months of treatment (T6), and at the end of treatment (after 12 months—T12), whereas CTRL were sampled at T0. Since some patients discontinued the clinical intervention, blood samples were available for 40 patients at T6 and 34 at T12.

### 2.2. Clinical Assessment

The diagnosis of BPD was confirmed using the Structured Clinical Interview for DSM-IV-TR disorders I and II [27,28]. At baseline, patients and CTRL underwent a multidimensional evaluation to assess various clinical features by scoring: (a) emotional dysregulation through the Difficulties in Emotion Regulation Scale (DERS) [29]; (b) BPD symptomatology through the Zanarini Rating Scale for borderline personality disorder (ZAN-BPD) [30]; (c) interpersonal functioning through the Inventory of Interpersonal Problems (IIP) [31]; in patients, this assessment was repeated at T6 and T12. At baseline, both patients and CTRL were also evaluated for the attachment style by the Attachment Style Questionnaire—Italian Version (ASQ) [32] and childhood traumatic experiences by the Childhood Trauma Questionnaire (CTQ) [33].

### 2.3. Intervention

Patients were randomly assigned to two intervention groups: MIT [34] and SCM [35]; among the 49 BPD patients enrolled, 25 underwent MIT and 24 SCM.

MIT is a cognitive behaviour-based psychotherapeutic approach aimed at increasing metacognitive abilities in order to improve personality functioning and promote better interpersonal relationships, following a goal hierarchy: (a) the assessment of symptoms, mental state, metacognitive functions, interpersonal problems, and emotion regulation skills; (b) intervention on the aspects that interfere with therapeutic alliance and/or threaten patient’s life (in particular, self-harming/suicidal behaviours); (c) intervention on symptoms that cause suffering to the patient; (d) intervention to promote integration of different mental states [34].

SCM belongs to the group of generalist models for the treatment of BPD and has been used as a comparator treatment against which other specialized protocols could be tested in research studies [35]. Therefore, SCM did not originally represent a type of psychotherapy but has then evolved into a distinct set of organizational psychotherapy principles [36]. SCM reflects the “best general psychiatric treatment for BPD”, and it is intended to be used by generalist mental health clinicians with minimal additional training. SCM is tailored to BPD symptomatology and employs a supportive approach with case management and advocacy support, whose core elements include psychoeducation, problem-solving, explicit safety planning, medication review, and assertive follow-up if appointments are missed [4,35].

Both treatments consist of a weekly 50 min individual session and a 90 min group session (manualised metacognitive skill training in MIT and problem-solving in SCM), covering a period of about 6 months during the year of individual psychotherapy.

When needed, BPD patients received pharmacological treatment, prescribed following a standardized protocol based on relevant pharmacological guidelines. The pharmacological agents included antidepressants, antipsychotics, anxiolytics, and mood stabilizers. During the clinical trial, substantial stability of drug therapy was maintained. Detailed information on pharmacological treatment has been previously reported in the original clinical trial [4].

Patients were classified as full responders if they showed a decrease of at least 25% in the DERS scores from T0 to T12 (corresponding to 36 points, i.e., a decrease of 1 score point for each of the 36 DERS items) and as partial responders if they showed a decrease of at least 12.5% (i.e., between 18 and 36 points). If the decrease was <12.5%, or if the DERS scores increased, they were classified as non-responders. These thresholds are those used in the original clinical trial [4].

### 2.4. Serum Collection and β-Endorphin Dosage

Peripheral venous blood samples were collected in the morning, after an overnight fast. Serum separation occurred after 1 h by centrifugation at 1680× *g* for 15 min. Serum samples were aliquoted and stored at −80 °C until the time of analysis.

β-endorphin concentrations were determined using the competitive enzyme-linked immunosorbent assay (ELISA) kit for Beta-Endorphin CEA806Hu (Cloud-Clone Corp., Katy, TX, USA), following the manufacturer’s instructions. Although assays were performed in different batches, all samples from the same patient across different time points, together with matched controls, were assayed within the same batch. Each sample was assessed in duplicate. The kit detection range is 1.6–1000 pg/mL, and the minimum detectable dose is less than 0.7 pg/mL. The intra-assay variability, expressed as the coefficient of variation, was <10% and the inter-assay variability <12%.

The laboratory analyses were performed blinded to treatment response.

### 2.5. Statistical Analyses

Data are described as mean ± standard deviation (SD) for continuous variables or as frequencies and percentages for categorical ones. For variables that were not normally distributed, data were additionally expressed as medians and interquartile ranges (IQRs). Gaussianity assumption for continuous variables was assessed by the Kolmogorov–Smirnov and Shapiro–Wilk tests, combined with visual inspection.

Comparisons between groups for continuous variables were carried out through Student’s t-tests for normally distributed variables and Mann–Whitney tests for non-normally distributed ones; for categorical variables, chi-square tests were applied. Correlations were evaluated through Pearson’s r coefficient or Spearman’s ρ coefficient depending on data normality.

For the longitudinal evaluation of clinical scores and β-endorphin levels, generalized linear mixed models were applied by setting time (three time points: T0, T6, and T12), treatment type group (MIT vs. SCM) or response group (Responders vs. Non-responders) and time × treatment type group/time × response group interaction as fixed effects, while a subject-specific random intercept was included to account for repeated measurements within subjects. Post hoc pairwise comparisons were adjusted for multiple testing using the Bonferroni correction.

Statistical significance was set at *p* < 0.05, and statistical analyses were carried out by SPSS 26.0 and through R language v.4.3.2 with the package “glmmTMB” for GLMM. Graphs were generated using GraphPad Prism v.8.4.3.

## 3. Results

### 3.1. Demographic and Clinical Characteristics of the Study Cohort

Descriptive statistics for the demographic and clinical features of BPD patients (n = 49) and CTRL (n = 25) are shown in Table 1, along with the results of the corresponding statistical tests comparing the two groups. BPD patients and CTRL subjects did not differ significantly in terms of age or sex distribution (*p* = 0.273 and *p* = 0.800, respectively). As expected, patients scored significantly higher than controls on the DERS, ZAN-BPD, Inventory of Interpersonal Problems (IIP), and Attachment Style Questionnaire (ASQ). The only exception was the ASQ subscale “Relationships as Secondary” for which the difference approached statistical significance (*p* = 0.054).

No significant differences were observed between patients in the MIT and SCM treatment groups for all the variables analyzed.

### 3.2. Case–Control Comparison of β-Endorphin Levels and Other Baseline Evaluations

Baseline β-endorphin levels were not normally distributed (Kolmogorov–Smirnov test: *p* = 0.003; Shapiro–Wilk test: *p* < 0.001); thus, non-parametric tests were applied.

**Figure 1:**
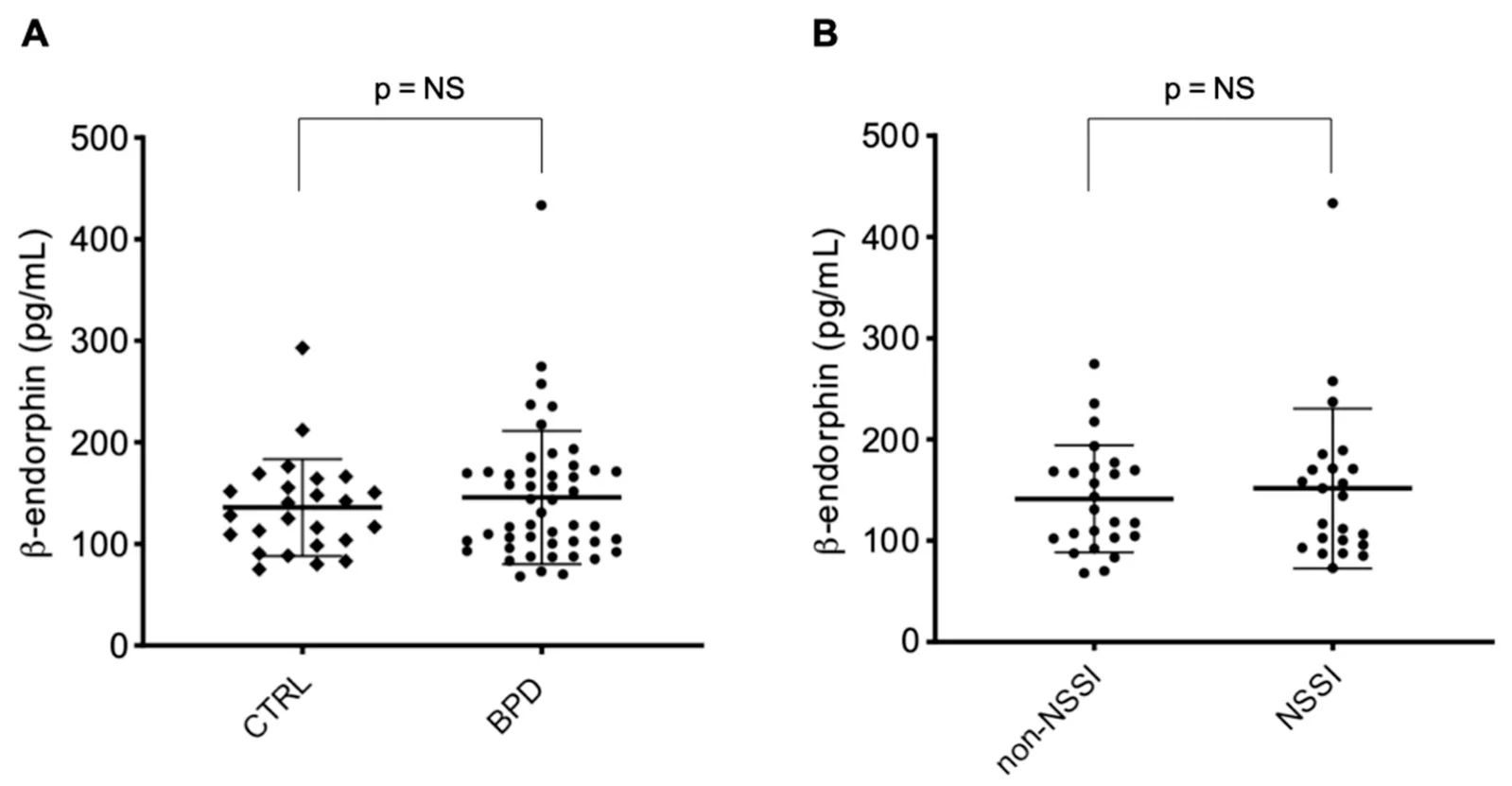
Baseline β-endorphin serum levels (expressed as pg/mL) did not differ significantly between patients with borderline personality disorder (BPD, n = 49) and unaffected controls (CTRL, n = 25) (panel (**A**), *p* = 0.587), nor between NSSI (n = 23) and non-NSSI (n = 25) BPD patients (panel (**B**), *p* = 0.959).

When comparing BPD patients vs. CTRL, no significant differences in serum β-endorphin levels were detected (BPD: 145.8 ± 65.6 pg/mL (median = 130.9, IQR = 70 pg/mL), CTRL: 136.0 ± 47.7 pg/mL (median = 127.9, IQR = 59 pg/mL); *p* = 0.587) (Figure 1A).

Moreover, β-endorphin concentrations did not correlate with disease severity, evaluated both by DERS and ZAN-BPD scales (*p* = 0.387 and *p* = 0.692, respectively), and with all the IIP and ASQ scores/subscores. In addition, β-endorphin levels did not significantly differ between patients receiving and not receiving psychotropic drugs (*p* = 0.725), nor between patients with or without a history of suicide attempt(s) (*p* = 0.936).

In light of the altered pain sensitivity reported in BPD and of the role of β-endorphin in pain perception, we also examined potential differences in its serum levels between patients who exhibited non-suicidal self-injury (NSSI, n = 23) and patients who did not (non-NSSI, n = 25; one patient was excluded from this analysis because of missing NSSI data). As shown in Figure 1B, no significant difference was found between groups (NSSI: 151.7 ± 78.9 pg/mL (median = 144.5, IQR = 75 pg/mL), non-NSSI: 141.6 ± 53.0 pg/mL (median = 130.9, IQR = 69 pg/mL); *p* = 0.959).

### 3.3. Outcomes of Psychotherapy Treatment and β-Endorphin Serum Levels

Among the entire cohort of 49 patients who participated in the study, 36 completed the 12-month treatment period. Overall, psychotherapy significantly reduced patients’ symptomatology, as evidenced by significant decreases in both DERS and ZAN-BPD scores over the course of treatment (*p* = 0.004 and *p* < 0.001, respectively). The mean DERS scores were 124.3 ± 25.0 at T0, 111.6 ± 28.3 at T6 and 109.7 ± 26.6 at T12; the mean ZAN-BPD scores were 15.9 ± 4.5 at T0, 11.7 ± 4.2 at T6 and 8.9 ± 4.0 at T12.

In detail, seven patients out of 36 (19.4%) were classified as full responders, and eight (22.2%) as partial responders, whereas twenty-one (58.3%) were considered non-responders to psychotherapy. For subsequent analyses, a binary classification was adopted, combining full and partial responders into the responder group (RESP, n = 15) and comparing them with non-responders (NON-RESP, n = 21). The MIT and SCM groups were comparable in terms of the distribution of RESP and NON-RESP patients (*p* = 0.821).

Importantly, the RESP and NON-RESP groups did not significantly differ in demographic or clinical variables at baseline, including age, sex, alcohol or substance abuse, psychotropic medication use, presence of NSSI, and clinical scale scores (Supplementary Table S1).

Concerning β-endorphin, a GLMM with a Gamma distribution and log link was fitted to account for the positively skewed distribution of the outcome; therefore, no direct log transformation of the raw data was applied. The model converged successfully, and diagnostics revealed no significant violations. Estimated marginal means were computed on the link scale and then back-transformed to the original response scale for interpretation.

Baseline β-endorphin concentrations did not differ significantly between the MIT and SCM groups.

**Figure 2:**
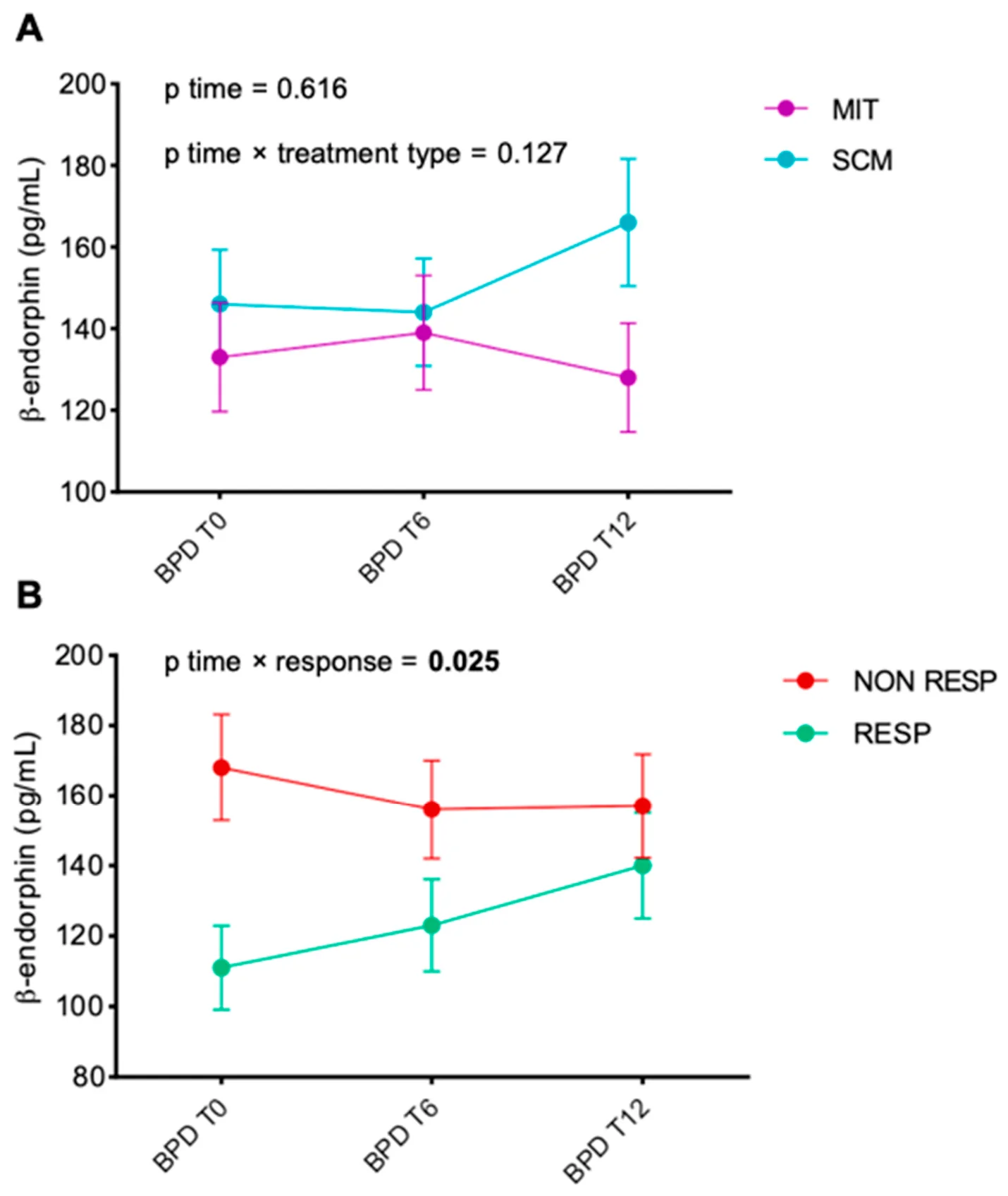
Longitudinal trajectory of β-endorphin serum levels (expressed as pg/mL) during treatment in BPD patients, from baseline (T0, n = 49) to 6 months (T6, n = 40) and 12 months (T12, n = 34) of treatment (end of treatment). β-endorphin concentrations did not show significant changes over time (*p* = 0.616). No significant interaction was observed with treatment type (panel (**A**), MIT vs. SCM, *p* = 0.127), whereas a significant interaction was identified with treatment response (panel (**B**), RESP vs. NON-RESP, *p* = 0.025).

Moreover, β-endorphin levels did not show significant changes over the treatment period (time effect: *p* = 0.616), nor was a significant time × treatment type interaction detected (*p* = 0.127) (estimated marginal means ± standard errors of the mean: MIT T0 = 133 ± 13.4 pg/mL, MIT T6 = 139 ± 14.0 pg/mL, MIT T12 = 128 ± 13.4 pg/mL; SCM T0 = 146 ± 13.4 pg/mL, SCM T6 = 144 ± 13.2 pg/mL, SCM T12 = 166 ± 15.6 pg/mL). The results are represented in Figure 2A.

We subsequently explored whether β-endorphin levels differentially changed in relation to treatment response. Given the absence of significant differences between the MIT and the SCM groups, the entire sample was considered, taking into account the treatment response status.

At baseline, β-endorphin concentrations significantly differed between the RESP and NON-RESP groups (*p* = 0.001), prompting further analyses of baseline β-endorphin as a potential predictor of treatment response (see the subsequent paragraph “Baseline β-endorphin as predictor of response to psychotherapy”).

When evaluating time × response interactions, a clear divergence emerged between RESP and NON-RESP patients (overall time × response interaction, *p* = 0.025), primarily driven by differences between the T0 and T12 assessments (adjusted *p* = 0.021). In particular, β-endorphin concentrations increased significantly in RESP patients from T0 to T12 (adjusted *p* = 0.016), whereas no significant variation was detected in NON-RESP patients over the same interval (adjusted *p* = 1.000).

The estimated marginal means ± standard errors were: RESP T0 = 111 ± 11.9 pg/mL, RESP T6 = 123 ± 13.2 pg/mL, RESP T12 = 140 ± 15.0 pg/mL; NON-RESP T0 = 168 ± 15.2 pg/mL, NON-RESP T6 = 156 ± 14.1 pg/mL, NON-RESP T12 = 157 ± 14.8 pg/mL. The results are represented in Figure 2B.

We also tested possible correlations between changes, from T0 to T12, in β-endorphin levels and corresponding changes in clinical scale scores. No significant result was observed, either in the whole patients’ cohort (correlation with Δ DERS: *p* = 0.086; correlation with Δ ZAN-BPD: *p* = 0.593) or when considering the responder group only (correlation with Δ DERS: *p* = 0.841; correlation with Δ ZAN-BPD: *p* = 0.719).

### 3.4. Baseline β-Endorphin as Predictor of Response to Psychotherapy

**Figure 3:**
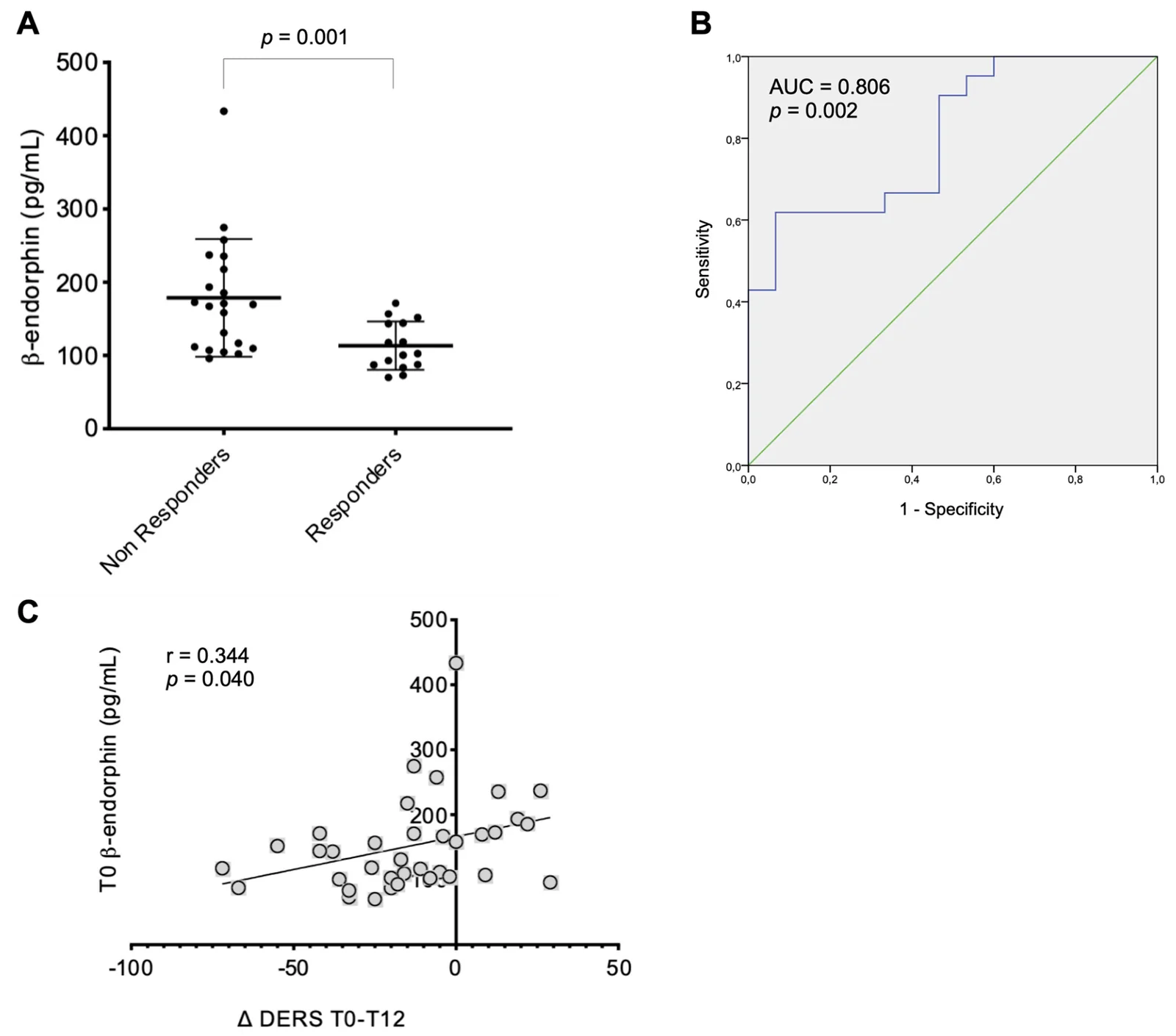
(**A**) Baseline β-endorphin serum levels (expressed as pg/mL) were significantly lower in responder vs. non-responder BPD patients (*p* = 0.001). (**B**) Exploratory ROC analysis indicated an AUC of 0.806 in discriminating between responders and non-responders (*p* = 0.002; the diagonal line represents the reference line for random classification). (**C**) A significant positive correlation was observed between baseline β-endorphin serum levels and Δ DERS, calculated as DERS T12—DERS T0; lower Δ DERS indicate a greater clinical improvement) (*p* = 0.040, r = 0.344).

Patients who responded to treatment exhibited significantly lower β-endorphin serum concentrations at T0 than non-responders (RESP: 113.53 ± 32.80 pg/mL (median = 102.6, IQR = 57 pg/mL), NON-RESP: 178.78 ± 80.05 pg/mL (median = 169.8, IQR = 116 pg/mL); *p* = 0.001), suggesting that lower baseline β-endorphin are associated with a more favourable treatment outcome (Figure 3A).

Receiver operating characteristic (ROC) curve analysis also demonstrated that baseline β-endorphin levels discriminated responders from non-responders to psychotherapy with good accuracy, yielding an area under the curve (AUC) of 0.806 and showing statistical significance (standard error = 0.072; 95% CI: 0.665–0.948, *p* = 0.002) (Figure 3B).

Although changes in β-endorphin concentrations during treatment did not correlate with changes in clinical severity, baseline β-endorphin levels positively correlated with Δ DERS scores, defined as DERS at T12—DERS at T0 (*p* = 0.040, r = 0.344) (Figure 3C). Since lower Δ DERS values indicate greater improvement in emotional regulation (or a smaller worsening, if positive), this finding indicates that lower baseline β-endorphin concentrations were associated with a more pronounced clinical improvement in emotional regulation. No corresponding correlation was observed for Δ ZAN-BPD scores (*p* = 0.938).

## 4. Discussion

In this study, we investigated the relationship between peripheral β-endorphin concentrations, clinical features and response to psychotherapy in patients with BPD. Baseline β-endorphin concentrations did not differ between patients with BPD and unaffected controls, nor were they associated with clinical severity measures, suicide attempts or NSSI. Moreover, psychotherapy was not accompanied by a significant overall change in β-endorphin concentrations across the entire cohort of patients. However, longitudinal analyses revealed a significant time × response interaction, indicating distinct trajectories according to treatment response. Specifically, β-endorphin levels increased significantly during treatment in responder patients, whereas they remained stable in non-responders.

A further relevant finding was that baseline serum β-endorphin emerged as a potential predictor of treatment response. In particular, responder patients exhibited lower β-endorphin concentrations at baseline than non-responders, and ROC analysis indicated good discriminatory ability in this exploratory analysis (AUC > 0.8). Consistently, lower baseline β-endorphin levels were associated with greater improvement in emotional regulation, as indexed by larger reductions in DERS scores over the course of treatment. By contrast, no correlation was found between changes in β-endorphin concentrations during treatment and either DERS or ZAN-BPD scores.

To our knowledge, this is the first study comparing peripheral β-endorphin levels between patients with BPD and unaffected controls. Previous studies have primarily investigated β-endorphin in relation to NSSI rather than BPD itself, leaving the question of whether altered β-endorphin represents a disorder-specific feature unexplored. We found no significant case–control difference and did not observe any association of baseline peripheral β-endorphin concentrations with baseline clinical symptoms, including NSSI. These findings suggest that peripheral β-endorphin levels are unlikely to represent a trait marker of BPD or to reflect the severity of its clinical manifestations.

With respect to NSSI, previous studies have yielded heterogeneous findings. In a study by Stanley and colleagues, adults with Cluster B personality disorders (mainly BPD, but also including antisocial, narcissistic and histrionic personality disorders) and a history of suicidal behaviour were stratified according to the presence or absence of repetitive NSSI. The authors found lower CSF concentrations of β-endorphin and met-enkephalin, another endogenous opioid, in individuals with NSSI compared with those without NSSI, suggesting a possible dysregulation of the endogenous opioid system in relation to self-injurious behaviours; however, the sample size was quite small (14 NSSI vs. 15 non-NSSI). Based on these findings, the authors proposed an opioid deficiency model, suggesting that reduced endogenous opioid tone may contribute to NSSI by increasing vulnerability to emotional distress and promoting self-injury as a mechanism to restore opioid homeostasis [21]. Similarly, two studies in adolescent populations reported reduced plasma β-endorphin levels in individuals exhibiting NSSI compared with controls, suggesting that reduced peripheral opioids may represent a biological correlate of NSSI, at least during adolescence. In particular, Van der Venne and colleagues investigated a relatively large cohort of female adolescents (94 with NSSI and 35 without NSSI). In addition to lower plasma β-endorphin concentrations, adolescents with NSSI showed increased pain thresholds and lower pain intensity ratings [25]. Cakin Memik and colleagues investigated a mixed-sex adolescent sample including 49 individuals with NSSI and 39 controls. Whereas lower plasma β-endorphin levels were observed in adolescents with NSSI compared with controls, no significant differences were found for met-enkephalin. Importantly, the authors highlighted that basal peripheral measurements may be influenced by several confounding factors and may not fully capture the dynamic changes in the endogenous opioid system. They suggested that repeated, time-dependent assessments of peripheral opioid neuropeptides in relation to NSSI episodes may provide a more accurate characterization of their involvement in self-injurious behaviours [24]. This consideration may also be relevant for interpreting our findings, as peripheral β-endorphin was assessed under basal conditions and not in temporal proximity to NSSI episodes or acute emotional states. Therefore, the absence of differences in basal β-endorphin levels between patients with and without NSSI does not exclude a possible involvement of opioid mechanisms in the regulation of self-injurious behaviours. More recently, Kao and collaborators also investigated plasma β-endorphin levels in adolescents (n = 53), examining possible associations with NSSI and BPD symptoms. In contrast to previous studies, no significant association was found between basal plasma β-endorphin concentrations and NSSI, nor with BPD symptoms [26]. These findings are consistent with our results, suggesting that peripheral β-endorphin levels may not reliably reflect the presence of self-injurious behaviours or borderline psychopathology. This is further supported by the study of Mürner-Lavanchy and colleagues, who applied a machine-learning approach to evaluate a panel of candidate biological markers, including β-endorphin, associated with NSSI in female adolescents. The strongest predictors of NSSI were low oxytocin, increased pain threshold and elevated leukocyte counts, whereas β-endorphin did not emerge as a relevant predictor [37].

Concerning the longitudinal assessment of β-endorphin changes during psychotherapy, to our knowledge no previous study has investigated this aspect in patients with BPD. Although in the present study psychotherapy was not associated with an overall change in β-endorphin concentrations, we observed a significant time × response interaction, indicating divergent trajectories according to treatment response. Specifically, besides showing lower baseline β-endorphin concentrations than non-responders, responders exhibited a significant increase in β-endorphin levels from baseline to treatment completion, whereas non-responders displayed no significant change over time. Regarding the increase in β-endorphin levels observed exclusively among responders we hypothesize that clinical improvement may be associated with greater adaptability of the endogenous opioid system. This finding could indicate that some patients are more capable of engaging opioid-related mechanisms during psychotherapy, potentially facilitating therapeutic response. Conversely, the absence of such changes in non-responders may reflect reduced capacity for an adaptive modulation of the endogenous opioid system in response to psychotherapy. This speculative interpretation should be considered together with the possibility of a ceiling effect: higher baseline β-endorphin concentrations may limit the extent of further increases over time. Overall, the observed pattern could suggest that alterations in endogenous opioid system do not characterize patients with BPD as a whole but may instead reflect biological heterogeneity among patients, potentially associated with differences in psychotherapy outcomes. However, these mechanistic interpretations remain speculative and require further investigation. Nevertheless, the predictive value of baseline β-endorphin for treatment response should be considered preliminary, given the small subgroup sizes (15 responders and 21 non-responders) and the relatively wide confidence interval of the ROC analysis (0.665–0.948). Notably, no other baseline variable, including indices of initial symptom severity, showed a significant association with treatment response; thus, the ability of baseline β-endorphin levels to distinguish between responders and non-responders may be of particular interest, given that clinical characteristics did not appear to differentiate the two groups.

Some limitations should be considered when interpreting the study findings. First, the relatively modest sample size, particularly the smaller subgroups in the longitudinal and treatment response analyses, limits the statistical power and the generalizability of the observed results. Second, the analyses examining baseline β-endorphin levels as a potential predictor of psychotherapy response were exploratory, and all the analyses were conducted without a validation cohort and should therefore be interpreted cautiously. In addition, the baseline group comparisons and correlation analyses were exploratory and were conducted without adjustment for multiple testing. Although this approach was considered appropriate given the hypothesis-generating nature of these analyses, the number of comparisons performed increases the possibility of type I error. Larger independent cohorts are therefore required to clarify the role of peripheral β-endorphin in the mechanisms underlying response to psychotherapy in BPD and to determine whether its baseline levels may serve as a possible predictive biomarker of treatment outcome.

## 5. Conclusions

In conclusion, in the present study, peripheral β-endorphin levels did not appear to be associated with BPD diagnosis or specific clinical manifestations such as NSSI but were associated with response to psychotherapy. These findings suggest that peripheral β-endorphin may represent a potential candidate biological marker associated with psychotherapy response, rather than a stable trait marker of BPD. However, its possible role in treatment responsiveness and its predictive value for psychotherapy outcome remain preliminary and warrant further investigation in larger samples and independent validation cohorts.

## Figures and Tables

**Table 1:** Demographic and clinical characteristics of the patient and control groups at baseline and results of the corresponding statistical comparisons.

	BPD Patients (n = 49)	CTRL (n = 25)	*p*-Value *
Sex (females vs. males)	40 (81.6%)	21 (84.0%)	0.800
Age (years; mean ± SD)	30.4 ± 8.0	28.3 ± 6.2	0.273
History of alcohol abuse (yes vs. no)	23 (46.9%)		
History of substance abuse (yes vs. no)	28 (57.1%)		
Psychotropic drug treatment (yes vs. no)	32 (65.3%)		
CTQ score (mean ± SD)	57.5 ± 16.9	32.1 ± 4.8	**<0.001**
DERS score (mean ± SD)	121.5 ± 25.8	63.0 ± 11.9	**<0.001**
ZAN-BPD score (mean ± SD)	15.5 ± 4.6	1.9 ± 1.3	**<0.001**
IIP Interpersonal sensitivity score (mean ± SD)	2.5 ± 0.7	1.1 ± 0.6	**<0.001**
IIP Interpersonal ambivalence score (mean ± SD)	2.1 ± 0.8	0.7 ± 0.5	**<0.001**
IIP Aggression score (mean ± SD)	2.0 ± 1.0	0.4 ± 0.4	**<0.001**
IIP Need for social approval score (mean ± SD)	2.4 ± 0.9	1.3 ± 0.7	**<0.001**
IIP Lack of sociability score (mean ± SD)	2.2 ± 1.0	0.8 ± 0.7	**<0.001**
IIP mean scores (mean ± SD)	2.2 ± 0.7	0.8 ± 0.4	**<0.001**
ASQ Confidence score (mean ± SD)	26.4 ± 6.4	32.8 ± 3.4	**<0.001**
ASQ Discomfort with closeness score (mean ± SD)	40.1 ± 6.0	32.9 ± 3.9	**<0.001**
ASQ Relationships as secondary score (mean ± SD)	17.6 ± 6.4	14.4 ± 6.1	0.054
ASQ Need for approval score (mean ± SD)	29.3 ± 6.7	19.1 ± 5.0	**<0.001**
ASQ Preoccupation with relationships score (mean ± SD)	36.1 ± 6.9	25.8 ± 5.8	**<0.001**

* The *p*-value refers to the comparison between BPD patients and CTRL. Statistically significant *p*-values are shown in bold.

## Data Availability

The data obtained in the study cannot be made publicly available for privacy and ethical reasons and are available on request from the corresponding author.

## Supplementary Materials

The following supporting information can be downloaded at: https://www.mdpi.com/article/10.3390/biology15211912/s1, Table S1: demographic and main clinical characteristics of the responder and non-responder groups at baseline and results of the corresponding statistical comparisons.

## References

[1] Bohus M., Stoffers-Winterling J., Sharp C., Krause-Utz A., Schmahl C., Lieb K. (2021). Borderline personality disorder. Lancet.

[2] Leichsenring F., Heim N., Leweke F., Spitzer C., Steinert C., Kernberg O.F. (2023). Borderline Personality Disorder: A Review. JAMA.

[3] Magni L.R., Carcione A., Ferrari C., Semerari A., Riccardi I., Nicolo’ G., Lanfredi M., Pedrini L., Cotelli M., Bocchio L. (2019). Neurobiological and clinical effect of metacognitive interpersonal therapy vs structured clinical model: Study protocol for a randomized controlled trial. BMC Psychiatry.

[4] Rossi R., Corbo D., Magni L.R., Pievani M., Nicolò G., Semerari A., Quattrini G., Riccardi I., Colle L., Conti L. (2023). Metacognitive interpersonal therapy in borderline personality disorder: Clinical and neuroimaging outcomes from the CLIMAMITHE study-A randomized clinical trial. Personal. Disord..

[5] Leichsenring F., Fonagy P., Heim N., Kernberg O.F., Leweke F., Luyten P., Salzer S., Spitzer C., Steinert C. (2024). Borderline personality disorder: A comprehensive review of diagnosis and clinical presentation, etiology, treatment, and current controversies. World Psychiatry.

[6] Thomas N., Gurvich C., Hudaib A.-R., Gavrilidis E., Kulkarni J. (2019). Systematic review and meta-analysis of basal cortisol levels in Borderline Personality Disorder compared to non-psychiatric controls. Psychoneuroendocrinology.

[7] Chiavetto L.B., Tardito D., Galbiati C., Ferrari C., Lanfredi M., Pedrini L., Carcione A., Riccardi I., Nicolò G., Rossi R. (2025). Reduction of oxytocin plasma levels in borderline personality disorder and normalization induced by psychotherapies. Psychol. Med..

[8] di Giacomo E., Andreini E., Santambrogio J., Arcara A., Clerici M. (2024). The interplay between borderline personality disorder and oxytocin: A systematic narrative review on possible contribution and treatment options. Front. Psychiatry.

[9] Ferreira A.C., Osório F.d.L. (2022). Peripheral oxytocin concentrations in psychiatric disorders—A systematic review and methanalysis: Further evidence. Prog. Neuropsychopharmacol. Biol. Psychiatry.

[10] Schmahl C., Baumgärtner U. (2015). Pain in Borderline Personality Disorder. Mod. Trends Pharmacopsychiatry.

[11] Bresin K., Gordon K.H. (2013). Endogenous opioids and nonsuicidal self-injury: A mechanism of affect regulation. Neurosci. Biobehav. Rev..

[12] Kleindienst N., Bohus M., Ludäscher P., Limberger M.F., Kuenkele K., Ebner-Priemer U.W., Chapman A.L., Reicherzer M., Stieglitz R.-D., Schmahl C. (2008). Motives for nonsuicidal self-injury among women with borderline personality disorder. J. Nerv. Ment. Dis..

[13] Ludäscher P., Greffrath W., Schmahl C., Kleindienst N., Kraus A., Baumgärtner U., Magerl W., Treede R., Bohus M. (2009). A cross-sectional investigation of discontinuation of self-injury and normalizing pain perception in patients with borderline personality disorder. Acta Psychiatr. Scand..

[14] Schloss N., Shabes P., Kuniss S., Willis F., Treede R., Schmahl C., Baumgärtner U. (2019). Differential perception of sharp pain in patients with borderline personality disorder. Eur. J. Pain.

[15] Bekrater-Bodmann R., Chung B.Y., Richter I., Wicking M., Foell J., Mancke F., Schmahl C., Flor H. (2015). Deficits in pain perception in borderline personality disorder: Results from the thermal grill illusion. Pain.

[16] Schmahl C., Greffrath W., Baumgärtner U., Schlereth T., Magerl W., Philipsen A., Lieb K., Bohus M., Treede R.-D. (2004). Differential nociceptive deficits in patients with borderline personality disorder and self-injurious behavior: Laser-evoked potentials, spatial discrimination of noxious stimuli, and pain ratings. Pain.

[17] Schmahl C., Bohus M., Esposito F., Treede R.-D., Di Salle F., Greffrath W., Ludaescher P., Jochims A., Lieb K., Scheffler K. (2006). Neural correlates of antinociception in borderline personality disorder. Arch. Gen. Psychiatry.

[18] Ludäscher P., Bohus M., Lieb K., Philipsen A., Jochims A., Schmahl C. (2007). Elevated pain thresholds correlate with dissociation and aversive arousal in patients with borderline personality disorder. Psychiatry Res..

[19] Magerl W., Burkart D., Fernandez A., Schmidt L.G., Treede R.-D. (2012). Persistent antinociception through repeated self-injury in patients with borderline personality disorder. Pain.

[20] Johnson B.N., McKernan L.C., Bruehl S. (2022). A Theoretical Endogenous Opioid Neurobiological Framework for Co-occurring Pain, Trauma, and Non-suicidal Self-injury. Curr. Pain Headache Rep..

[21] Stanley B., Sher L., Wilson S., Ekman R., Huang Y., Mann J.J. (2010). Non-suicidal self-injurious behavior, endogenous opioids and monoamine neurotransmitters. J. Affect Disord..

[22] Prossin A.R., Love T.M., Koeppe R.A., Zubieta J.-K., Silk K.R. (2010). Dysregulation of regional endogenous opioid function in borderline personality disorder. Am. J. Psychiatry.

[23] Pilozzi A., Carro C., Huang X. (2020). Roles of β-Endorphin in Stress, Behavior, Neuroinflammation, and Brain Energy Metabolism. Int. J. Mol. Sci..

[24] Memik N.C., Hunc F., Kalayci S., Demir N., Senturk E., Gundogdu O.Y., Dillioglugil M.O. (2023). Assessment of Plasma-Endogenous Opioid Neuropeptide Levels and Psychometric Properties of Non-Suicidal Self-Injury in Adolescents. Arch. Suicide Res..

[25] van der Venne P., Balint A., Drews E., Parzer P., Resch F., Koenig J., Kaess M. (2021). Pain sensitivity and plasma beta-endorphin in adolescent non-suicidal self-injury. J. Affect. Disord..

[26] Kao H.-T., Mürner-Lavanchy I., Lerch S., von Stosch E., Berger T., Koenig J., Kaess M. (2024). Longitudinal associations between beta-endorphin, nonsuicidal self-injury and comorbid psychopathology. Psychiatry Res..

[27] First M.B., Spitzel R.L., Gibbon M., Williams J.B.W. (1994). Structured Clinical Interview for DSM-IV Axis II Personality Disorders (SCID-II).

[28] First M.B., Spitzer R.L., Gibbon M., Williams E.J.B.W. (1995). The Structured Clinical Interview for DSM-III-R Personality Disorders (SCID-II). Part I: Description. J. Personal. Disord..

[29] Gratz K.L., Roemer L. (2004). Multidimensional Assessment of Emotion Regulation and Dysregulation: Development, Factor Structure, and Initial Validation of the Difficulties in Emotion Regulation Scale. J. Psychopathol. Behav. Assess..

[30] Zanarini M.C. (2003). Zanarini Rating Scale For Borderline Personality Disorder (ZAN-BPD): A Continuous Measure of DSM-IV Borderline Psychopathology. J. Personal. Disord..

[31] Pilkonis P.A., Kim Y., Proietti J.M., Barkham M. (1996). Scales for Personality Disorders Developed from the Inventory of Interpersonal Problems. J. Personal. Disord..

[32] Fossati A., Feeney J.A., Donati D., Donini M., Novella L., Bagnato M., Acquarini E., Maffei C. (2003). On the Dimensionality of the Attachment Style Questionnaire in Italian Clinical and Nonclinical Participants. J. Soc. Personal. Relatsh..

[33] Bernstein B.P., Fink L.A. (1998). Childhood Trauma Questionnaire: A Retrospective Self-Report.

[34] Carcione A., Nicolo G., Semerari A. (2021). Complex Cases of Personality Disorders: Metacognitive Interpersonal Therapy.

[35] Bateman A., Fonagy P. (2009). Randomized controlled trial of outpatient mentalization-based treatment versus structured clinical management for borderline personality disorder. Am. J. Psychiatry.

[36] Edmonds J. (2020). Structured Clinical Management (SCM). EFPT Psychotherapy Guidebook.

[37] Mürner-Lavanchy I., Koenig J., Reichl C., Josi J., Cavelti M., Kaess M. (2024). The quest for a biological phenotype of adolescent non-suicidal self-injury: A machine-learning approach. Transl. Psychiatry.

