# Supplementary material for: Peripheral β-Endorphin in Borderline Personality Disorder: Investigating Clinical Correlates and Longitudinal Changes During Psychotherapy
Source: Biology (Basel). 2026 Oct 22;15(21):1912. doi: 10.3390/biology15211912 (PMC13761840; doi:10.3390/biology15211912)
Supplement: Supplementary file 1 [file biology-15-01912-s001.zip › biology-4558349-supplementary.pdf]

Supplementary Table S1: demographic and main clinical characteristics of the responder and non-responder groups at baseline and results of the corresponding statistical comparisons.

	**Responders (n=15)**	**Non responders (n=21)**	**p-value**
Sex (females vs. males)	12 (80.0%)	17 (81.0%)	0.943
Age (years; mean ± SD)	30.3 ± 6.7	30.6 ± 9.0	0.918
History of alcohol abuse (yes vs. no)	8 (53.3%)	10 (47.6%)	0.735
History of substance abuse (yes vs. no)	10 (66.7%)	12 (57.1%)	0.563
Psychotropic drug treatment (yes vs. no)	11 (73.3%)	11 (52.4%)	0.207
Presence of non-suicidal self injury (yes vs. no)	8 (53.3%)	9 (42.9%)	0.535
DERS score (mean ± SD)	131.7 ± 20.2	118.9 ± 27.1	0.130
ZAN-BPD score (mean ± SD)	15.6 ± 3.4	16.0 ± 5.3	0.775
